# High Co-Infection Rate of *Trichomonas vaginalis* and *Candidatus* Mycoplasma Girerdii in Gansu Province, China

**DOI:** 10.3390/healthcare9060706

**Published:** 2021-06-10

**Authors:** Shuhui Xu, Zhixin Wang, Hang Zhou, Yongfeng Fu, Meng Feng, Xunjia Cheng

**Affiliations:** 1 Department of Medical Microbiology and Parasitology, School of Basic Medical Sciences, Fudan University, Shanghai 200032, China; 17111010072@fudan.edu.cn (S.X.); 17111010070@fudan.edu.cn (Z.W.); 18111010069@fudan.edu.cn (H.Z.); yffu@fudan.edu.cn (Y.F.); 2 Department of Medical Microbiology and Parasitology, Medical College of He Xi University, Zhangye 734000, China

**Keywords:** *Trichomonas vaginalis*, *Candidatus* mycoplasma girerdii, *Mycoplasma hominis*, genetic characteristics, co-infection, symbiosis

## Abstract

*Trichomonas vaginalis* (*Tv*) is a flagellated protist parasite that infects the human urogenital tract. The symbiotic relationship between *Tv* and *Mycoplasma hominis* has been reported. Recent studies have identified a new *Mycoplasma* strain, *Candidatus* Mycoplasma girerdii (*Ca.* M. girerdii), present in the vaginal secretions of women and have shown that this strain may be related to trichomoniasis. Here, we evaluated the presence of *Tv*, *M. hominis* and *Ca.* M. girerdii in 312 clinical samples from adult women diagnosed with vaginitis in Zhangye, Gansu province, China. Among these samples, 94, 153, and 48 were *Tv*, *M. hominis* and *Ca.* M. girerdii positive, respectively. Moreover, *Tv* was highly frequent in 17–30-year-old women in this region. Forty samples (83.3%) positive for *Ca.* M. girerdii were also positive for *Tv*. Six *Tv* isolates were successfully cultured, including five isolates that showed symbiotic relationships with *Mycoplasma*. This is the first report to evaluate the genetic characteristics of *Ca.* M. girerdii in China and may therefore provide insights into the effects of *Ca.* M. girerdii on the reproductive health of women.

## 1. Introduction

Trichomonas vaginalis (*Tv*) is the causative agent of trichomoniasis. This organism adapts to the surroundings of the human urogenital tract and causes trichomoniasis and urethritis. *Tv* is considered a nonviral sexually transmitted infection (STI) and is responsible for almost 2.5 billion annual cases of STIs worldwide [1]. The World Health Organization estimated that the global prevalence of *Tv* in 2016 was approximately 156 million cases, accounting for almost half of the global burden of STIs during that year [2]. Trichomoniasis is a sex-related infection that mainly affects women. The infection is often asymptomatic, and when present, its symptoms can range widely from itching to burning, dyspareunia, congestion of the vaginal mucosa or cervix, and malodorous discharge [1].

In recent years, various studies have shown that *Tv* has developed a symbiosis with several sexually transmitted pathogens. In particular, the symbiotic relationship between *Mycoplasma* and *Tv* has attracted much interest among researchers [1]. Indeed, the symbiosis between *Tv* and *Mycoplasma hominnis* was the first described between two obligated pathogens responsible for human parasitic diseases [3]. *Tv* has been shown to undergo symbiosis with *M. hominnis*, which can be present in up to 53% of asymptomatic females [4]. The co-infection rate of *M. hominnis* with *Tv* has been shown to range from 20% to over 90% [5,6,7,8]. The presence of *M. hominnis* synergistically upregulates the pro-inflammatory response in human macrophages exposed to *Tv* [9]. Moreover, a recent study identified a new *Mycoplasma* species, which shares an association with *Tv*. Initially, this species was termed “Mnola” and was found to be 85% identical to the closest human-associated species (*M. genitalium* and *M. pneumoniae*) and 94% identical to the closest cloned sequences [10]. Fettweis et al. used a metagenomic strategy to identify and expand Mnola and proposed the genome sequences of four independently identified strains [11]. As a result, the genome was named *Candidatus* Mycoplasma girerdii (*Ca.* M. girerdii) [11]. A considerable number of *Tv*-infected women have a unique vaginal microbiota, including a predominance of *Ca.* M. girerdii [10]. Moreover, a previous study reported that 54% of *Tv* isolates harbored *Mycoplasma* spp., and phylogenetic analysis revealed the newly identified species to be *Ca.* M. girerdii (59.3%) [12,13].

In this study, we report the detection and genetic characteristics of *Ca*. M. girerdii in China for the first time. These findings provide a theoretical basis for future research on the pathogenicity of *Tv* and *Ca*. M. girerdii anda may facilitate analyses of *Ca*. M. girerdii as an essential symbiotic organism compared with other *Mycoplasma* spp.

## 2. Materials and Methods

### 2.1. Collection of Clinical Specimens

We collected 312 clinical samples from adult women (17–75 years of age) with vaginitis between July and October 2018. Women who attended the Affiliated Zhangye People’s Hospital of the Medical College of He Xi University and were diagnosed with vulva pruritus accompanied by gray-white foamy leucorrhea, which was suspected to be caused by *Tv* infection, were enrolled in this study. Vaginal swabs were collected during routine pelvic examinations; one sample was obtained from each patient. No written informed consent was required from the patients because the study was deemed exempt. All samples were anonymized. The Institutional Ethics Committees of Affiliated Zhangye People’s Hospital of Medical College of He Xi University approved the study protocol (Ethics approval no.: 20180703).

### 2.2. Extraction of Genomic DNA

Vaginal swab specimens were collected from women with pruritus vulvae and gray-white foamy leucorrhoea, which was suspected to be caused by *Tv* infection. Each vaginal swab was divided into two parts and each was placed in a 2-mL saline tube. One tube was used for wet mount and culture, the other tube was immediately stored at −20 °C for PCR diagnosis. Total genomic DNA was extracted using a DNeasy Blood and Tissue kit (Tiangen, Beijing, China) according to the manufacturer’s experimental procedures and methods [14].

### 2.3. Polymerase Chain Reaction (PCR) Amplification

Primers were designed to target the conserved regions of the 18S ribosomal gene of *Tv* and the 16S rRNA gene of *Mycoplasma*. Table 1 shows the primer sequences used in this study. *Tv*18S and *Tv*18AS primers were designed according to the full-length sequence of the *Tv* 18S rRNA gene (accession no.: U17510). Mh16S2S and Mh16S2AS primers were designed according to the full-length sequence of the *M. hominis* 16S rRNA gene (accession no.: M96660). Furthermore, Mg16S-423F/Mg16S-736R and Mg207F/Mg1408R primers were also designed according to the *Ca*. M. girerdii 16S rRNA gene (accession no.: CP007711). After specific identification and sequencing, the designed *Ca*. M. girerdii primers were confirmed as targeting the *Ca*. M. girerdii gene by BLAST search. PCR was performed using a thermal cycler Gene Amp PCR system (Bio-Rad Laboratories, Hercules, CA, USA).

Standard PCR was conducted in a total volume of 50 μL. The amplification procedure included 3 min of denaturation at 94 °C, followed by 35 cycles of denaturation for 15 s at 94 °C, 30 s of annealing (temperatures are shown in Table 1), and 30 s of extension (or 90 s for primers Mg207F and Mg1048R) at 72 °C. A final extension step at 72 °C for 7 min was also included in each cycle [14].

### 2.4. Parasites and Culture Conditions

Clinical samples of *Tv* were collected and examined by wet mount; samples positive for *Tv* trophozoites were cultured in Diamond’s TYM medium supplemented with 10% horse serum (Gibco, Grand Island, NY, USA), penicillin (250 U/mL), streptomycin (250 μg/mL), and amphotericin B (50 μg/mL) (Thermo Fisher Scientific, Waltham, MA, USA). After subculturing 5 times, the antibiotics were successively decreased to penicillin (50 U/mL), and streptomycin (50 μg/mL).

### 2.5. Confocal Microscopy

Indirect immunofluorescence assays were used to assess the location of *Mycoplasma* in *Tv* cells. *Tv* trophozoites were gently washed with phosphate-buffered saline (PBS), fixed with 4% paraformaldehyde in PBS, and permeabilized in 0.1% Triton X-100 (Amresco, Solon, OH, USA). *Mycoplasma* cells were detected by incubating with anti-*M. hominis* p120 monoclonal antibodies (H37S) (Thermo Fisher Scientific), followed by incubation with Alexa Fluor 488 goat anti-mouse IgG1 (H + L) antibodies (Abcam, Cambridge, UK). Confocal images were acquired using a confocal microscope (Leica TCS SP8; Leica, Wetzlar, Germany).

### 2.6. Data Analysis and Statistics

SPSS 21 statistical software was used to analyze epidemiological data. Associations of the positive rate of *Tv* with patient age and living area were analyzed using regression analysis. *Tv* infection and co-infection rates were analyzed using χ^2^ tests.

A BLAST analysis was performed against a local database. The database contained 60 *Ca.* M. girerdii 16S rRNA gene sequences, which were previously detected in vaginal specimens by us or by other researchers, as reported in the literature. All sequences were initially aligned using ClustalW multiple alignments in BioEdit software. A phylogenetic tree was established using the Test Neighbor-Joining Tree settings in MEGA-X software [15].

## 3. Results

### 3.1. Epidemiological Characteristics of Trichomonas Vaginalis

We collected vaginal swab specimens from women with pruritus vulvae and gray-white foamy leucorrhoea, which was suspected to be caused by *Tv* infection. In total, 94 *Tv*-positive samples were obtained from 312 patients with vaginitis, yielding a positive rate of 30.1% for *Tv* infection. Notably, patient’s age was found to play a significant role in the onset of *Tv*. Additionally, there were 105 patients with vaginitis and 44 (41.9%) patients positive for *Tv* infection between the ages of 17 and 30 years (*p* = 0.011; Table 2).

### 3.2. Co-Infection Rate of Tv and Various Mycoplasma

Sequence analysis of all *Mycoplasma* PCR products isolated from 312 samples revealed that 48 samples (15.4%) harbored *Ca*. M. girerdii and 153 (49%) samples harbored *M. hominis*. Notably, 40 samples (83.3%) that were positive for *Ca*. M. girerdii were also positive for *Tv*. Co-infections of *M. hominis*/*Tv* was detected in 52.3% (80/153) of cases. In all 218 *Tv*-negative samples, eight and 73 were positive for *Ca*. M. girerdii and *M. hominis*, respectively. This observation indicated that *Ca*. M. girerdii (*p* < 0.001) and *M. hominis* (*p* < 0.001) were significantly associated with *Tv* infection (Table 3).

### 3.3. Tv Gene Analysis

In this study, 18S rDNA gene sequences of six Zhangye *Tv* samples were sequenced and used in multiple sequences alignments. The Zhangye isolates of the sequenced *Tv* 18S rDNA gene were identical to those of the Henan isolates (KM603328, KM603336), the Philippines isolates (KM282385, JX943583), the Iran isolates (KX061402, KX061408), the USA isolate (EU215370), and the Vienna isolate (AY338476). These findings indicated that the Zhangye population of *Tv* parasites showed the same level of genetic diversity.

### 3.4. Ca. M. Girerdii Gene Analyses

**Figure 1:**
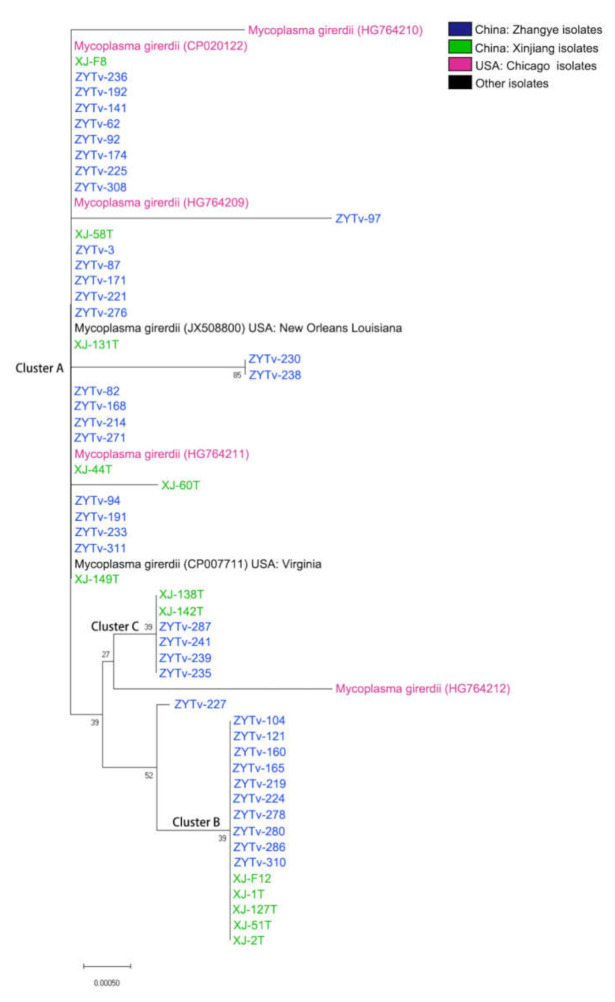
NJ tree analysis of 16S rDNA gene sequences showing the phylogenetic position of the Zhangye *Ca*. M. girerdii gene se-quence within all *Ca*. M. girerdii gene sequences reported in GenBank (Xinjiang samples were collected by Liu Jun in our laboratory). The tree was derived using 16S rDNA gene sequences (approximately 1200 nucleotides) obtained from GenBank. Original values are shown at the branch points. Gene accession numbers and sample source locations are shown after the organism names included in the tree.

In this study, we obtained 48 *Ca.* M. girerdii 16S rDNA gene sequences. In total, 59 *Ca.* M. girerdii 16S rDNA gene sequences were used to generate a phylogenetic tree (Figure 1). The *Ca.* M. girerdii gene sequences included those from 39 Zhangye isolates and 13 Xinjiang isolates. The same gene sequence of seven isolates obtained from different sources could be divided into three clusters: A, B, and C. The Zhangye *Ca.* M. girerdii isolates are most closely associated with all genes that have been reported with similar genetic distances among isolates from Xinjiang (Figure 1). The Zhangye *Ca.* M. girerdii 16S rDNA gene sequences were submitted to the Genebank database (access number: LC554417 to LC554421).

### 3.5. Mycoplasma Localized in Tv Cells

**Figure 2:**
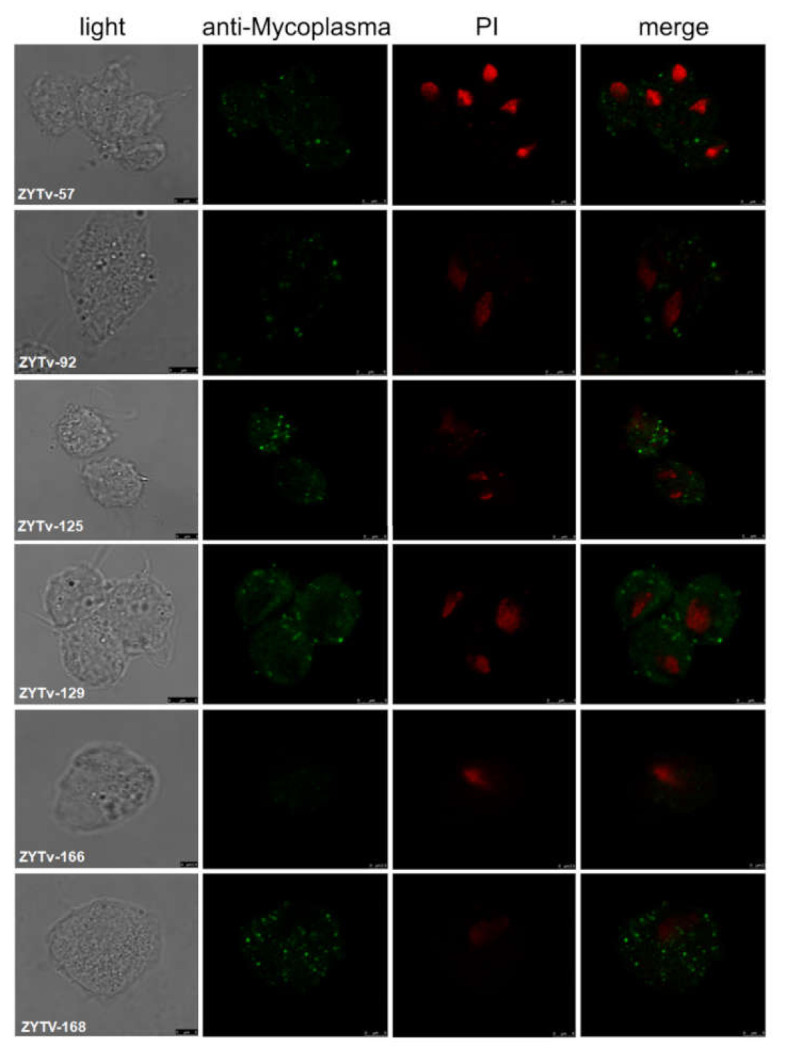
The cellular localization of *M. hominis* in *Tv* isolates. Four panels represent the same area of a protozoan monolayer immunostained as described in Materials and Methods. Light: surface image of *Tv*; anti-*Mycoplasma*: FITC fluorescence showing intracellular *M. hominis* (green); PI: images of the nucleus of *Tv* after PI staining (red); merge: superimposed panels of anti-*M. hominis* and PI.

In this study, six *Tv* isolates were successfully cultured. In addition, *Mycoplasma* symbiosis was detected by PCR (primers in Table 1) in these isolates at first and at every month for 6 months of continuous culture. One isolate, ZYTv-92, had both *M. hominis* and *Ca*. M. girerdii symbiosis. The classifications of *Mycoplasma* symbiosis for six isolates are listed in Table 4. The cellular localization of *M. hominis* in the trophozoites of *Tv* after 6 months of continuous culture was evaluated, and the results suggested that the clinical samples ZYTv-57, ZYTv-92, ZYTv-125, ZYTv-129, and ZYTv-168 exhibited *M. hominis* symbiosis (Figure 2).

## 4. Discussion

The estimated global prevalence of new trichomoniasis cases was 143 million among women ages 15–49 years in 2012 [2]. Moreover, *Tv* infection rates are relatively high (8.5%) in community samples from young women of reproductive age in India [16], and *Tv* infection prevalence has been estimated to be 0.5% among men and 1.8% among women aged 18–59 years in the USA, with the highest rates among black men (4.2%) and women (8.9%) [17]. Additionally, the prevalence of *Tv* infection is approximately 9.9% in rural China [18], and in Iran, the highest and lowest percentages of *Tv* infection in women were observed in patients 45–50 and 20–30 years old, respectively [19]. In this study, we found that the positive rate of *Tv* in patients with vaginitis was 30.1% in Zhangye, Gansu province, China; the high positive rate of *Tv* in this region may be related to the presence of vaginitis in the patients included in this study.

In our study, we found that *Tv* was highly prevalent in women between the ages of 17 and 30 years in our study area; this may be related to the increased sexual activity of women during this period [20]. A recent study found *Tv* to be significantly associated with having two or more sexual partners in the past year. The concurrency of sexual relationships and the number of sexual partners may differ by occupation, residence, and education level [21].

Multiple sequence comparison of the *Tv* 18S rDNA gene demonstrated that there was low genetic diversity among the *Tv* isolates. However, moderate-to-high genetic differentiation between isolates from different sites was observed in *Tv* isolates from Northwest China, which should be considered a single population. Moreover, the *Tv* gene from Zhangye isolates was mainly identical, and homology was similar to that in samples from the Philippines and Henan. These findings further confirmed that all *Tv* isolates originated from a common ancestor [14,22].

Studies of the symbiosis of pathogenic bacteria with protozoan vectors have provided insights into the symbiosis of organisms, although the exact mechanisms remain unknown. Indeed, the symbiotic relationship between *Mycoplasma* and *Tv* has attracted much attention [1], and researchers have been particularly interested in evaluating the symbiosis of *M. hominis* with *Tv* [12]. Symbiosis between *Tv* and *M. hominis* has been extensively studied and is observed in up to 90% of *Tv* clinical isolates in Europe [23]. Several groups used PCR to demonstrate the presence of *M. hominis* in *Tv* isolates of different geographic origins, with infection rates ranging from 5% to over 89% [1]. Moreover, *Ca*. M. girerdii has recently been shown to be associated with trichomonads identified in the vagina by researchers using next-generation sequencing [10]. In this study, *M. hominis* and *Ca*. M. girerdii were strongly associated with *Tv* in the examined population. Thus, *Ca*. M. girerdii may be another specific *Mycoplasma* species that can cause co-infections with *Tv*. Our results indicate that *Ca*. M. girerdii exhibits strong and unique associations with *Tv*. To the best of our knowledge, this is the first study to report the occurrence of *Ca*. M. girerdii in China.

Our findings were consistent with two previous reports on the prevalence of *Ca*. M. girerdii [10,11]. Accordingly, *Tv* may be the driving force behind infection with *Ca*. M. girerdii, and this transformation of the vaginal environment may enhance its causative force [12,24]. Notably, *Ca*. M. girerdii genome sequencing highlighted the presence of putative virulence genes, such as hemolysin, endopeptidase, and collagenase [25]. *Ca*. M. girerdii seems to lack gluconeogenesis and the tricarboxylic acid cycle, similar to other *Mycoplasma* species. Moreover, the arginine dihydrolase pathway and urease genes are absent from *Ca*. M. girerdii, implying that *Ca*. M. girerdii cannot utilize arginine and urea. This may be the reason for *Ca*. M. girerdii dependence on *Tv* [11,25].

The phylogenetic tree of *Mycoplasma* 16S rDNA gene sequences showing the phylogenetic position of the Zhangye *Ca*. M. girerdii gene sequence within all *Mycoplasma* gene sequences reported in GenBank demonstrated that the *Ca*. M. girerdii gene of Zhangye isolates was mainly concentrated in the same cluster. This species-specific relationship is most likely related to the genetic characteristics of *M. genitalium* and uncultured *Mycoplasma* spp. These results were consistent with studies of this uncultivated species, and the organism showed 85% identity with the closest human-associated species, *M. genitalium* [24].

## 5. Conclusions

Overall, our findings demonstrated a high co-infection rate between *Tv* and *Mycoplasma* in Zhangye, Gansu Provence, China. *Ca*. M. girerdii and *M. hominis* were the most closely related *Mycoplasma* species showing co-infections with *Tv* in Zhangye. These findings provide important insights and further our understanding of the impact of *Ca*. M. girerdii on the reproductive health of women. Additionally, Zhangye *Ca*. M. girerdiiisolates were most closely associated with strains from the USA and Xinjiang. Clarifying the reasons for the strong and unique association between *Ca*. M. girerdii and *Tv* is likely to have wider implications for the epidemiology and disease control of trichomoniasis.

## Figures and Tables

**Table 1:** Primers used in this study.

Gene or Locus	Sequence (5′–3′)	Product (bp)	AT (°C)
Tv18S	ATCAGAGGCACGCCATTC	580	60
Tv18AS	CGCCCTTGATCGACAGAA		
Mh16S2S	GGCTAATGCCGGATACGC	330	60
Mh16S2S	GGTACCGTCAGTCTGCAAT		
Mg16S-423F	TTTATTAGGGACGAACGGCACT	313	55
Mg16S-736R	CAGTTGTGACCTAAGTTCTCGC		
Mg207F	TTTAGGATGAGGGTGCGGTTTA	1201	55
Mg1408R	TAGCAGGACGGTTTTAGGTATT		

**Table 2:** Age distribution of *Trichomonas vaginalis* infection among all patients with vaginitis.

Age (years)	Total Patients (*n* = 312)	Patients with *Tv* Infection (*n* = 94) (30.1%)	*p*-Value	OR (95% CI)
17–30	105	44 (41.9%)	0.011	3.275 (1.319–8.134)
31–40	62	12 (19.4%)	0.781	1.159 (0.410–3.274)
41–50	106	31 (29.2%)	0.128	2.054 (0.813–5.189)
51–75	39	7 (17.9%)		1

Abbreviations: CI, confidence interval; OR, odds ratio.

**Table 3:** Characteristics of *Tv*+ women (cases) and *Tv*—women (controls).

Total Samples (*n* = 312)				
		*Mycoplasma* Positive		χ^2^ *p*-Value
*Mycoplasma* positive rate		*Tv* positive (*n* = 94)	*Tv* negative (*n* = 218)	
*Ca*. M. girerdii	48 (15.4%) *	40 (83.3%) **	8	<0.001
*M. hominis*	153 (49%) *	80 (52.3) **	73	<0.001

*: Percentage in total samples; **: Percentage in *Mycoplasma*-positive samples.

**Table 4:** *Mycoplasma*-symbiotic (+) and *Mycoplasma*-free (−) in *Tv* isolates.

*Tv* Isolates	Type of *Mycoplasma* Symbiosis	
	*M. hominis*	*Ca*. M. Girerdii
ZYTv-166	−	−
ZYTv-57	+	−
ZYTv-92	+	+
ZYTv-125	+	−
ZYTv-129	+	−
ZYTv-168	+	−

## Data Availability

The data presented in this study are available on request from the corresponding author.
